# Detection to Disruption: A Comprehensive Review of Bacterial Biofilms and Therapeutic Advances

**DOI:** 10.3390/antibiotics15040396

**Published:** 2026-04-13

**Authors:** Pranay Amruth Maroju, Angad S. Sidhu, Amogh R. Motaganahalli, Robert E. Minto, Fatih Zor, Christine Kelley-Patteson, Rahim Rahimi, Aladdin H. Hassanein, Mithun Sinha

**Affiliations:** 1 Department of Surgery, Indiana University School of Medicine, Indianapolis, IN 46202, USA; pamaroju@iu.edu (P.A.M.); sidhua@iu.edu (A.S.S.); amotagan08@gmail.com (A.R.M.); fzor@iu.edu (F.Z.); ahassane@iu.edu (A.H.H.); 2 Department of Chemistry and Chemical Biology, Indiana University, Indianapolis, IN 46202, USA; rminto@iu.edu; 3 Meridian Plastic Surgeons, Indianapolis, IN 46290, USA; ckelleypatteson@me.com; 4 School of Materials Engineering, Purdue University, West Lafayette, IN 47907, USA; rrahimi@purdue.edu; 5 Birck Nanotechnology Center, Purdue University, West Lafayette, IN 47907, USA

**Keywords:** bacterial biofilms, extracellular polymeric substance, antibiotic resistance, chronic infections, device-associated infections, breast implant illness, biofilm detection, biofilm therapeutics

## Abstract

Bacterial biofilms are structured microbial communities enclosed within a self-produced extracellular polymeric substance matrix composed of polysaccharides, proteins, extracellular DNA, and lipids. This matrix promotes adhesion, structural stability, and the development of heterogeneous microenvironments that restrict antimicrobial penetration and shield bacteria from host immune responses. As a result, biofilms are major contributors to chronic, recurrent, device-related, and difficult-to-treat infections, posing a major challenge for clinical management and antimicrobial stewardship. This review summarizes current understandings of biofilm biology, its clinical relevance, including the stages of biofilm development, the composition and protective roles of the matrix, and the physiological heterogeneity that arises during maturation. It also examines key mechanisms underlying biofilm tolerance and resistance, such as limited antibiotic diffusion, and sequestration, enzymatic inactivation, efflux pump upregulation, persister cell formation, and horizontal gene transfer. In addition, it highlights important clinical settings in which biofilms are implicated, including cystic fibrosis, chronic wounds, osteomyelitis, implant- or device-associated infections, and breast implant illness, in which persistent implant-associated biofilms and the resulting chronic inflammatory milieu have been hypothesized to contribute to local and systemic manifestations in a subset of patients. The review further discusses conventional and emerging approaches for biofilm detection alongwith real-time monitoring. Biofilm-associated infections remain difficult to eradicate because persistence is driven by multiple interconnected protective mechanisms. Effective management therefore requires integrated strategies that combine accurate detection with multifaceted therapies, including antibiotics alongside matrix-disrupting enzymes, quorum-sensing inhibitors, bacteriophages, metabolic reactivators, and nanotechnology-based delivery systems. Advances in multi-omics and system-level modeling will be essential for developing next-generation strategies to prevent, monitor, and treat biofilm-associated disease.

## 1. Introduction

Microbial biofilms are structured communities of bacteria that adhere to living tissues or abiotic surfaces and become embedded in a self-produced extracellular polymeric substance matrix (EPS) [1,2,3]. The bacteria in biofilm interact with one another [4], and the biofilm matrix endows beneficial emergent properties that cannot be seen in planktonic microorganisms [5,6]. The matrix stabilizes adhesion, retains water and biomolecules, and creates local gradients in oxygen, nutrients, pH, and antimicrobial exposure, generating subpopulations with distinct physiological states [7,8]. This organization is clinically important because its collective properties help explain why biofilms are strongly associated with persistent infection, immune evasion, and poor therapeutic response in clinical settings [9,10].

The persistence of biofilms is particularly challenging because antibacterial failure in these communities arises from mechanisms beyond resistance alone [11,12]. Resistance refers to the inherited or stably acquired ability of microorganisms to grow in the presence of antimicrobial concentrations that inhibit susceptible populations [13], usually reflected by an increased minimal inhibitory concentration (MIC) [14], and mediated by mechanisms such as mutation or acquisition of resistance genes [11,15]. Tolerance, in contrast, is the capacity of a susceptible population to survive longer during antimicrobial exposure without a change in MIC [13,14]. Persistence describes a small phenotypic subpopulation of transiently dormant or very slow-growing cells that survive lethal drug concentrations and can repopulate once treatment stops [16]. In biofilms, restricted drug penetration, nutrient and oxygen limitation, stress responses, and metabolic heterogeneity favor persistence and tolerance [17,18,19]. It may also facilitate the emergence and maintenance of resistance during prolonged infection [11,20]. These mechanisms make biofilms highly relevant to the clinical course of chronic and relapsing infections [21].

Biofilms are implicated across major disease groups, including chronic wounds [22], airway infections [23], urinary tract infections (UTIs) [24], osteomyelitis [25], periodontitis [26], and a wide range of infections involving prosthetic joints [27], heart valves [28], breast implant illness (BII) [29,30] and other implanted materials. In these settings, antimicrobial therapy may temporarily reduce bacterial burden yet fail to eradicate the protected community, especially when an infected surface, foreign body, or devitalized tissue remains in place. This mismatch has important diagnostic and therapeutic implications. Standard susceptibility testing is performed mainly on planktonic cells and therefore may underestimate the survival capacity of biofilm-embedded organisms [31], which helps explain why apparently susceptible isolates may still be associated with treatment failure in chronic infections [31,32]. Similarly, superficial cultures may not reflect organisms embedded within mature biofilms, especially in chronic wounds [33] and device-associated infections [32], underscoring the need for improved sampling strategies and biofilm-aware diagnostics [31,34].

Biofilms constitute a major clinical challenge because they contribute to persistence, treatment failure, and recurrence across many chronic and device-associated infections. Frequently cited historical estimates suggest that biofilms are involved in about 65% of microbial infections and up to 80% of chronic infections [35], although these values should be interpreted as broad estimates rather than precise contemporary surveillance figures. In the United States, healthcare-associated infections have been estimated to affect approximately 1.7 million hospitalized patients annually and to contribute to nearly 99,000 deaths, underscoring the scale of infection burden in settings where device-associated biofilms are common [36]. Implanted and indwelling medical devices are especially vulnerable because microorganisms readily form biofilms on biomaterial surfaces, where they become difficult to eradicate and often necessitate device removal or exchange [37,38]. In chronic wounds, biofilms are common and are associated with persistent inflammation, delayed healing, and reduced response to antimicrobial therapy [39,40]. In cystic fibrosis (CF), chronic airway infection with biofilm-forming *Pseudomonas aeruginosa* is a prototypic example of biofilm-driven disease and a major cause of morbidity and treatment burden [41]. This burden is further compounded by antimicrobial resistance (AMR); a global analysis estimated that 4.71 million deaths (95% UI 4.23–5.19) were associated with AMR in 2021 [42,43]. Biofilm-grown bacteria can tolerate antibiotic concentrations orders of magnitude higher than their planktonic counterparts, in some models approaching about 1000-fold greater tolerance [44]. Together, these observations make clear that biofilm research is a major epidemiologic and therapeutic priority rather than a niche topic [37,38]. In this context, this review examines the clinical significance of bacterial biofilms, with particular emphasis on the mechanisms by which resistance, tolerance, and persistence undermine treatment and on why improved diagnostics and biofilm-targeted therapies are needed.

## 2. What Are Biofilms

### 2.1. Cellular Organization of Biofilms

Biofilms exhibit a high degree of cellular organization that distinguishes them from free-living planktonic microorganisms [5,38]. Rather than forming random aggregates, cells within a biofilm are arranged in structured microcolonies and interconnected communities embedded in an EPS matrix [45,46]. This spatial architecture creates localized microenvironments with gradients in oxygen, nutrients, pH, and metabolic byproducts, causing cells in different regions of the biofilm to adopt distinct physiological states [44]. Surface-associated cells may remain relatively active and proliferative, whereas deeper populations often become slow-growing, stress-adapted, or dormant [44,47]. Channels and voids within the biofilm further enhance organization by permitting the movement of water, nutrients, signaling molecules, and waste products [37,48]. In addition, close cell-to-cell proximity promotes quorum sensing, coordinated gene expression, metabolic cooperation, and horizontal gene transfer [5,49,50]. Together, this organized multicellular arrangement enables biofilms to function as dynamic, resilient communities with enhanced survival, persistence, and tolerance to antimicrobial and host-mediated stresses [5,38,47].

### 2.2. Matrix Composition

At the core of the matrix is a heterogeneous mixture of EPS, extracellular DNA (eDNA), proteins, lipids and small molecules [46]. The exact composition varies by species, strain, environmental conditions and developmental stage, but several common structural components are consistently observed [19,51]. Polysaccharides form the primary structural scaffold in many biofilms, but the dominant polymer differs between Gram-negative and Gram-positive bacteria [52]. In Gram-negative bacteria such as *P. aeruginosa*, polysaccharides including alginate, Psl and Pel impart unique adhesive, diffusive, and mechanical properties to the biofilm matrix [53,54]. Psl is a neutral branched penta-saccharide composed of D-mannose, D-glucose, and L-rhamnose and promotes surface attachment to substrates including plastic, glass, mucin, and epithelial cells [55,56,57]. In Gram-positive staphylococci, particularly *Staphylococcus epidermidis* and *Staphylococcus aureus*, biofilm accumulation is frequently associated with polysaccharide intercellular adhesin/poly-β-(1→6)-N-acetylglucosamine (PIA/PNAG) [58,59]. Proteinaceous matrices rich in surface adhesins and amyloid-like factors are also common depending on the strain and growth conditions [60,61,62]. The diversity of polysaccharide chemistry underlies species-specific matrix behaviors, from highly hydrated, mucoid matrices in alginate-rich biofilms to more hydrophobic, protein-rich matrices in others [63].

eDNA is another key matrix component that contributes to structural integrity and microbial interactions [64,65]. It acts as a polyanionic cross-linker that binds cations (Mg^2+^, Ca^2+^), bridging polysaccharides and proteins [65]. Chemically, it buffers ionic microenvironments and functionally promotes horizontal gene transfer by serving as a gene reservoir [66,67]. eDNA can originate from controlled secretion, autolysis of subpopulations [65], or host neutrophil extracellular traps (NETs) [68], and its presence has profound effects on matrix rheology and antibiotic interactions. For instance, positively charged aminoglycosides are sequestered by eDNA and anionic polysaccharides, reducing free drug concentration and limiting penetration [8,69].

Proteins, including functional amyloids and adhesins, contribute to structural reinforcement and cell–cell adhesion within biofilms [51,62]. Enzymes associated with the matrix (nucleases, proteases, β-lactamases) can actively remodel or inactivate incoming antimicrobials and modulate matrix composition over time [70,71]. Lipids and outer membrane vesicles (OMVs) also contribute to matrix organization by transporting enzymes, signaling molecules, and DNA while influencing hydrophobic interactions and molecular transport [72,73,74].

### 2.3. Architecture

Mature biofilms behave as viscoelastic materials that can withstand mechanical stress and exhibit both solid-like and fluid-like behavior depending on the magnitude and timescale of deformation [75]. Biofilms are structurally heterogeneous and commonly contain clustered microcolonies, water channels, and void spaces that facilitate nutrient transport and waste removal [76]. In clinical environments, biofilm architecture is also influenced by interactions with host-derived components and surface-associated molecules. Adhesins expressed by bacterial cells facilitate attachment to host tissues [77], while host macromolecules such as fibrin, mucins, collagen and lipids can become incorporated into the developing biofilm matrix, contributing to structural stability thereby shaping the spatial organization of microbial communities [78]. These structural features generate microscale gradients in oxygen, nutrients, pH, and metabolites, which in turn produce physiologically distinct subpopulations across the biofilm [44,79,80]. As a result, biofilms develop stratified metabolic zones in which microbial cells adopt different physiological states depending on their local microenvironment. These microenvironments also influence antimicrobial activity by altering drug penetration and bacterial physiological state [81,82].

### 2.4. EPS Matrix Function

The EPS matrix performs several critical functions within biofilms. The EPS matrix provides mechanical stability, mediates adhesion, and helps protect embedded cells from physical disruption and chemical stress [46,75]. Matrix polymers can retard or alter antimicrobial penetration, and electrostatic interactions involving matrix components such as eDNA and polysaccharides can sequester cations or positively charged antibiotics, thereby modifying antimicrobial susceptibility [66,81,83]. In some biofilms, matrix-associated β-lactamase activity can hydrolyze β-lactam antibiotics before they reach deeper bacterial cells [84]. Biofilm regulation also depends on quorum sensing and the intracellular second messenger cyclic-di-GMP, which coordinates matrix production, motility repression, maturation, and dispersal [85,86,87]. The close spatial arrangement of cells within biofilms also promotes metabolic cooperation and cross-feeding, particularly in polymicrobial communities [88,89]. These interactions allow biofilm communities to exploit diverse nutrient sources and persist under nutrient limitations or environmental stress.

## 3. The Biofilm Life Cycle and Host Defense in Clinically Relevant Microenvironments

**Figure 1:**
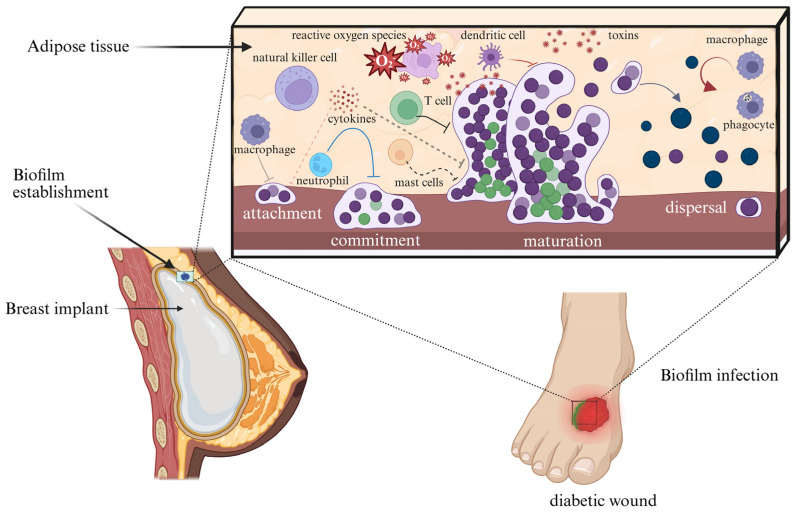
Biofilm development in peri-implant tissues and diabetic wounds along with immune barriers to clearance. Schematic overview of biofilm establishment and persistence in two clinically relevant microenvironments: peri-implant tissues (foreign-body surface and peri-implant niche) and diabetic/chronic wounds (damaged tissue with impaired perfusion). Initial attachment, followed by reversible adhesion and irreversible attachment of planktonic bacteria to implant material or exposed tissue. Microcolony formation and maturation into a structured biofilm encased in an extracellular polymeric substance (EPS) matrix with nutrient/oxygen gradients and phenotypic heterogeneity. Immune engagement (neutrophils, macrophages, natural killer cells and T cells) is blunted by physical shielding from the EPS, altered bacterial metabolism (slow growth/dormancy), and biofilm-driven immune modulation. Persistence and dissemination, including release of planktonic cells/aggregates during dispersal, drive recurrent infection and chronic inflammation.

Biofilm formation is a regulated, multistage process in which microbial adaptation and host defense are in constant opposition [37]. In peri-implant tissues and chronic or diabetic wounds, the stages of attachment, maturation, and dispersal are clinically important because they contribute to persistence, immune evasion, chronic inflammation, and treatment failure [90,91,92]. Although these stages are often presented linearly, biofilm development in vivo is dynamic and often cyclical. In implant settings, host proteins coating the biomaterial can enhance bacterial adhesion, whereas in diabetic wounds, ischemia, barrier dysfunction, and immune dysregulation impair early clearance [77,90,92]. At this stage, host defense is primarily preventive and includes intact epithelial barriers, antimicrobial peptides, complement, normal perfusion, and mechanical clearance [90,91,93]. During the transition to irreversible attachment, adhesins, pili, fimbriae, and rising cyclic-di-GMP levels promote stable adhesion, EPS production, and repression of motility [86,87,94]. If host clearance fails, the attached population matures into a three-dimensional community with water channels and steep gradients in oxygen, nutrients, and pH [44,79]. These gradients generate phenotypic heterogeneity, with more metabolically active cells near nutrient-replete regions and slower-growing or dormant cells in deeper zones [44,80]. Quorum sensing and other regulatory networks further refine virulence, matrix remodeling, stress responses, and dispersal behavior [85,86]. Clinically, the mature biofilm state is difficult to eradicate because matrix-mediated protection and physiological heterogeneity reduce susceptibility to antibiotics and host clearance [75,82,95]. Neutrophils and macrophages may remain abundant, but phagocytic killing is less effective, and unresolved inflammation can cause collateral tissue damage without sterilizing the infection [90,91,95]. This problem is amplified around implants by the foreign-body effect and in diabetic wounds by impaired perfusion and defective inflammatory resolution [90,92,93]. Dispersal is clinically important because it can seed recurrence and spread to adjacent sites [79,85]. In response to cues such as nutrient limitation, oxygen shifts, waste accumulation, nitric oxide signaling, or mechanical disturbance, biofilms can release single cells or aggregates through matrix degradation, erosion, or sloughing [79,85]. Host defenses attempt to clear these released cells, but this is often incomplete in compromised tissues [90,91,92]. Clinically relevant biofilm phenotypes are not confined to firmly surface-attached growth, because non-attached aggregates in chronic wounds and airway secretions can display biofilm-like physiology, tolerance, and persistence [96,97,98]. From a clinical perspective, the biofilm life cycle therefore reflects both microbial development and progressive failure of host clearance mechanisms [90,91,95]. Biofilm development and the major host-immune barriers to clearance in peri-implant tissues and diabetic wounds are summarized in Figure 1.

## 4. Antimicrobial Resistance

**Figure 2:**
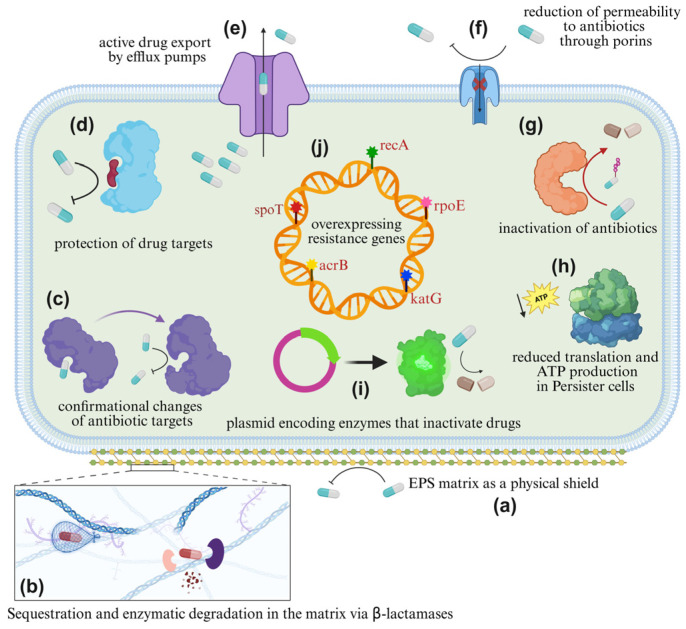
Mechanisms by which biofilms withstand antimicrobial stress and promote the emergence and spread of antimicrobial resistance (AMR). (**a**) The extracellular polymeric substance (EPS) matrix limits antimicrobial diffusion. (**b**) Antibiotics are sequestered within the matrix through binding to extracellular DNA (eDNA), polysaccharides, and other polymers, and may also be degraded by β-lactamases. (**c**,**d**) Bacteria may modify antibiotic targets through conformational changes or protect these targets from antibiotic action. (**e**) Biofilm cells upregulate efflux pumps that actively expel antibiotics. (**f**) Reduced permeability, including decreased uptake through porins, restricts antibiotic entry. (**g**) Enzymes further inactivate or degrade antimicrobial agents, lowering effective drug concentrations. (**h**) Persister cells exhibit reduced translation, low ATP production, and diminished metabolic activity, resulting in decreased susceptibility to antibiotic killing. (**i**) Horizontal gene transfer, including plasmid-mediated exchange of resistance determinants, promotes the emergence and dissemination of AMR. (**j**) Overexpression of resistance-associated genes further strengthens biofilm survival under antimicrobial stress.

Biofilm resilience to antimicrobials emerges from the interplay of physical protection, altered physiology, and genetic exchange [12,99,100]. Within the biofilm, bacteria are embedded in a dense extracellular polymeric matrix that impedes drug penetration, sequesters antimicrobial molecules, and buffers rapid changes in the local environment [11,101,102,103]. At the same time, steep gradients of nutrients, oxygen, pH, and waste products generate heterogeneous microenvironments that drive subpopulations into slow-growing, dormant, or persister states that are intrinsically less susceptible to many drugs [44,97,104]. Overlaying this is an elevated rate of genetic exchange via plasmids, mobile elements, and released DNA within the matrix, which promotes the spread and diversification of resistance determinants among neighboring cells [50,105,106]. Together, these mechanisms operate across spatial and temporal scales, from micron-scale niches to population-level dynamics, to produce a multi-layered tolerance phenotype that is reversible and context dependent, and therefore fundamentally distinct from classical planktonic antibiotic resistance, which is typically defined by stable, heritable changes in minimum inhibitory concentration measured in well-mixed, free-living cultures of bacteria [107,108,109]. Key biofilm-associated mechanisms that drive antimicrobial tolerance and the emergence/spread of AMR are summarized in Figure 2.

### 4.1. Diffusion–Reaction Barriers and Drug Sequestration

The EPS presents a diffusion–reaction barrier that reshapes antibiotic concentration profiles as drugs penetrate the biomass [100,103]. Electrostatic interactions and charge heterogeneity in the matrix can bind and sequester antibiotics (aminoglycosides binding to anionic eDNA/polysaccharides), reducing free drug concentrations in the deeper parts of the biofilm [110]. Meanwhile, matrix-associated enzymatic activities (β-lactamases, esterases) degrade susceptible antibiotics in situ before they reach target bacteria [84,111]. The combined effect is a lower effective concentration at the cellular level than predicted by bulk dosing. To better understand antibiotic penetration within biofilms, diffusion–reaction models have been developed to describe how antimicrobial molecules move through and interact with the EPS matrix. These models typically apply modified forms of Fick’s laws of diffusion, incorporating parameters such as biofilm thickness, matrix density, antibiotic binding affinity, and enzymatic degradation rates [103,112,113]. The resulting frameworks predict that antibiotic concentration decreases progressively with depth as molecules diffuse inward while simultaneously being adsorbed, inactivated, or consumed. Reaction–diffusion simulations therefore explain why deeper biofilm layers often experience sub-inhibitory drug concentrations despite high external dosing. Such modeling approaches provide quantitative insight into transport limitations within biofilms and help identify how matrix composition, biofilm architecture, and antibiotic physicochemical properties collectively determine drug accessibility to bacterial target sites during dosing [100,103].

### 4.2. Microenvironmental Effects on Drug Action

Local physicochemical heterogeneity within biofilms further reduces antimicrobial efficacy. Microdomains of altered pH, redox potential, oxygen tension, and metal ion availability develop because of restricted diffusion and intense local metabolism, creating conditions that differ markedly from the surrounding environment [44,83,114]. These gradients are not merely passive features of biofilm architecture. They directly influence drug penetration, uptake, stability, and intracellular activity. Local acidification can alter the ionization state of both antimicrobial agents and bacterial surfaces, thereby affecting membrane permeability and drug–target interactions [115]. At the same time, redox gradients shape cellular respiratory activity and membrane energetics, which are critical determinants of susceptibility for several antibiotic classes’ potential [116,117,118]. Ion chelation within the extracellular matrix, mediated in part by extracellular polymeric substances and extracellular DNA, can further modify antimicrobial potency by sequestering cations, destabilizing normal membrane interactions, and altering local chemical conditions [83].

Aminoglycosides provide a clear example of this phenomenon. Their uptake depends on an intact proton motive force (PMF) and membrane potential generated by active respiration [114,117]. In acidic, hypoxic, or anoxic regions of the biofilm, where energy production is reduced, aminoglycoside transport across the bacterial membrane is markedly impaired [104,114,119]. As a result, even when the drug reaches these deeper layers, intracellular accumulation may remain subtherapeutic. Similarly, oxygen depletion in the inner regions of biofilms drives a metabolic shift away from aerobic respiration toward fermentation or anaerobic respiration, producing physiological states in which antibiotics that are most effective against actively respiring cells lose potency [120]. These localized metabolic adaptations therefore create protected niches in which bacteria are not genetically resistant but are phenotypically far less susceptible to treatment [107,108]. Collectively, pH microdomains, redox stratification, and ion sequestration reinforce the capacity of biofilms to withstand antimicrobial exposure by converting a structurally complex community into a chemically and physiologically heterogeneous therapeutic barrier.

### 4.3. Slow Growth, Dormancy and Persister Cells

A hallmark of biofilms is metabolic heterogeneity; many cells in the interior adopt slow-growth or near-dormant states before or during antibiotic exposure [44,104,121]. These persister cells survive antibiotic exposure without heritable changes in MICs, but by virtue of low metabolic activity, reduced target engagement, and activated stress responses [104,107,122]. In biofilms, steep gradients of oxygen, nutrients, pH, redox state, and waste products expand these low-energy micro niches and favor multidrug tolerance [44,100]. Persisters arise through both stochastic phenotypic switching and environmentally induced programs [104,121,123]. Among the best characterized stress pathways is the stringent response, in which accumulation of (p)ppGpp under starvation, oxidative stress, envelope stress, immune pressure, or antibiotic exposure suppresses replication, transcription, and translation and shifts cells toward a protected survival state [124,125]. Toxin–antitoxin systems can further reinforce reversible growth arrest by inhibiting translation, DNA replication, or other core processes, although their contribution varies by species and experimental context. In biofilms, these programs intersect with quorum sensing and c-di-GMP signaling, which further support matrix production, stress adaptation, and dormancy-associated survival [123,126]. Once antibiotic pressure is removed, persisters can resuscitate, repopulate the biofilm, and drive relapse, helping explain why bactericidal drugs that mainly target active cellular processes often fail to eradicate established biofilm infections [122,127,128].

### 4.4. Biofilm-Specific Transcriptional Programs

Biofilm growth is accompanied by broad transcriptional reprogramming that shifts bacterial physiology from rapid planktonic proliferation to a protected, stress-adapted state [82,99,129]. Rather than depending on a single resistance determinant, biofilm-associated cells coordinately alter the expression of genes involved in transport, metabolism, cell envelope maintenance, oxidative stress defense, and damage repair [82,99]. These changes reduce intracellular antibiotic accumulation, diminish drug–target engagement, and improve survival during prolonged antimicrobial exposure. In *P. aeruginosa*, for example, the biofilm-associated regulator BrlR promotes tolerance by activating multidrug efflux systems, including PA1874–PA1877, thereby lowering intracellular antibiotic concentrations [130]. Biofilm cells also induce oxidative stress defenses, protein quality-control pathways, and DNA repair systems, allowing them to withstand both antibiotic-mediated injury and host-derived reactive species [131].

Comparable transcriptional adaptations occur in Gram-positive biofilms, although the specific regulators differ. In *S. aureus*, biofilm growth is associated with major changes in global regulatory networks, including the alternative sigma factor sigB, the agr quorum-sensing system, sarA, and two-component systems such as WalKR, ArlRS, and GraRS [132,133,134]. These pathways remodel expression of adhesins, autolysins, extracellular matrix-associated factors, and stress-response genes, while also enhancing tolerance to cell envelope stress, oxidative injury, and antimicrobial exposure. In many contexts, reduced agr activity favors a sessile, adhesive phenotype, whereas sigB and sarA support persistence by promoting stress tolerance and biofilm stability. In *S. epidermidis*, regulators linked to the ica operon and polysaccharide intercellular adhesin production similarly reinforced matrix accumulation and chronic device-associated persistence [135,136]. In enterococci, systems such as fsr and other stress-responsive regulators contribute to biofilm maturation, surface adherence, and survival under hostile conditions [137,138]. Across Gram-positive organisms, these transcriptional programs converge on a common outcome, reduced growth-associated vulnerability, strengthened envelopes and stress defenses, altered surface architecture, and improved capacity to survive antibiotic and immune attack.

Thus, biofilm-associated tolerance is not merely a passive consequence of matrix shielding or nutrient limitation [99]. It is also an active physiological state driven by regulatory circuits in both Gram-negative and Gram-positive bacteria. By coupling efflux, stress adaptation, repair, and biofilm-specific surface programs, these transcriptional networks create cells that are harder to kill, more resilient to host defenses, and more capable of sustaining chronic infection [129].

### 4.5. Horizontal Gene Transfer and Gene Flow

Dense, surface-attached communities facilitate horizontal gene transfer (HGT) via conjugative plasmids, bacteriophage-mediated transduction, and DNA transfer mediated by OMVs [50,105,106,139]. The EPS matrix concentrates DNA and mobile elements, boosting the probability of gene exchange. This ecological intimacy speeds up the dissemination of AMR genes and mobile genetic elements, integrating tolerance and resistance at the community level [50,65,140]. Biofilms are typically polymicrobial, bringing phylogenetically diverse bacteria into stable, long-term contact within the same matrix. This taxonomic diversity enlarges the pool of mobile genetic elements and broad-host-range plasmids present, increasing opportunities for interspecies conjugation, transduction, and transformation [105]. Metabolic cross-feeding and quorum-sensing within such mixed communities can further induce competence by stress responses, enhancing horizontal gene transfer and promoting the spread of AMR determinants across otherwise unrelated taxa [105,106].

### 4.6. Evolutionary Dynamics and Adaptive Mutation in Biofilms

Biofilms function as evolutionary niches where spatial structure and environmental heterogeneity accelerate the emergence of antibiotic-resistant variants [141]. Cells near the surface experience higher antibiotic exposure, while deeper cells often encounter nutrient limitation and metabolic stress [142]. These stresses activate global responses such as SOS and stringent responses, which can increase mutation rates through error-prone DNA polymerases and reduced DNA repair fidelity [141]. Oxidative stress further promotes DNA damage and mutagenesis [106]. Because biofilms restrict dispersal, beneficial mutations can expand locally within clonal microcolonies rather than competing with the entire population. Repeated cycles of stress and recovery therefore promote adaptive diversification and the emergence of stable antibiotic-resistant lineages [12,106].

### 4.7. Outer Membrane Vesicles and Phage Dynamics

Outer membrane vesicles serve as vehicles for DNA, proteins, and enzymes, shielding genetic material from nucleases and enabling the delivery of resistance determinants to recipient cells. In parallel, bacteriophages and prophage dynamics within biofilms also modulate gene flow and can propagate resistance or virulence phenotypes. These mobile elements blur the boundary between individual-level genetics and community-level adaptation [143]. Phages are often extraordinarily abundant within biofilms, where the EPS matrix traps viral particles and creates localized “hotspots” of phage–host interaction [144]. In this structured environment, temperate phages frequently adopt lysogenic lifestyles, integrating into bacterial genomes and acting as major vectors for transduction of AMR and virulence genes [145]. Phage predation imposes strong selective pressures that shape community composition, while induction of prophages and phage-mediated lysis can drive biofilm turnover, nutrient release, and dispersal of resistant subpopulations. Collectively, these processes generate a biofilm-specific tolerance phenotype in which standard planktonic minimum inhibitory concentrations fail to reflect true clinical resilience. Antimicrobials that appear effective against free-living cells often perform poorly against biofilm-embedded populations. Consequently, biofilm-centered metrics such as the minimum biofilm inhibitory concentration (MBIC) and the minimum biofilm eradication concentration (MBEC) more accurately capture antimicrobial efficacy in biofilm-associated infections [143].

## 5. Detecting and Monitoring Biofilms

Effective prevention and management of biofilm-associated infections in clinical settings rely on sensitive, specific, and context-appropriate detection tools that can identify persistent microbial communities contributing to chronic wounds, device-related infections, and antimicrobial resistance. No single assay addresses every aspect, given the heterogeneous nature of biofilms. Robust workflows therefore combine complementary methods such as phenotypic (e.g., tissue culture plate, Congo red agar), microscopic techniques exemplified by confocal laser scanning and scanning electron microscopy (SEM), molecular techniques (PCR-based assays, 16S rRNA Next generation sequencing), spectroscopic techniques such as near-infrared (NIR) imaging, and others like biofilm blotting to triangulate key parameters like biomass quantification, cell viability, 3D structure, matrix composition, and real-time dynamics. This integrated strategy enhances diagnostic accuracy and guides targeted therapies. A summary of clinically relevant detection and monitoring approaches, including advantages and limitations, is provided in Table 1.

### 5.1. Classical Laboratory Methods

Crystal violet staining remains one of the most widely used assays in biofilm research because it provides a simple measurement of total attached biomass in microtiter plates [97,155]. In the standard microtiter-plate format, attached biomass is stained with crystal violet, the retained dye is solubilized, and absorbance is measured spectrophotometrically as an indirect index of total biofilm mass [156,157]. Because the assay is easily scaled to 96-well or similar formats, it is well-suited to comparative screening of strains, growth conditions, mutants, and antibiofilm treatments [155,156]. Standardized optical-density cutoffs have also been used to classify isolates as weak, moderate, or strong biofilm producers, although the exact thresholds depend on the protocol and controls used [158,159]. Crystal violet is also commonly used in biofilm susceptibility workflows to quantify inhibition of biofilm formation or reduction in established biomass after treatment [31,160]. Colony-forming unit enumeration after mechanical or enzymatic disruption remains a standard method for quantifying the culturable, viable fraction of biofilm populations [155,161]. CFU-based assays are especially useful in antimicrobial testing because they directly measure surviving culturable bacteria, but they do not capture viable-but-nonculturable cells and require careful standardization of biofilm recovery and plating [155,161].

### 5.2. Microscopy and High-Resolution Structural and Chemical Characterization

Microscopy-based methods are central to biofilm research because they allow direct visualization of biofilm viability, architecture, matrix organization, and physicochemical heterogeneity [155,162]. Fluorescence-based viability assays are widely used for rapid assessment of intact biofilms, particularly when combined with epifluorescence microscopy, widefield fluorescence microscopy, or confocal laser scanning microscopy (CLSM) [162]. Common dyes include SYTO 9 to label total bacterial cells, propidium iodide to identify cells with compromised membranes, and DAPI for nucleic acid staining [163,164]. These assays exploit differences in membrane permeability and provide a practical means to examine live/dead distributions within intact biofilms. However, interpretation requires caution, since membrane compromise does not always indicate irreversible cell death, especially in slow-growing, injured, or dormant subpopulations [165].

Among optical imaging approaches, CLSM remains one of the most informative tools for biofilm analysis because it enables high-resolution, three-dimensional imaging of hydrated, minimally disturbed biofilms [162,166]. When combined with fluorescent probes targeting cells and extracellular matrix components, such as lectins for polysaccharides, nucleic acid dyes, fluorescently tagged antibodies, or genetically encoded fluorescent proteins, CLSM can resolve biofilm thickness, microcolony organization, spatial heterogeneity, and matrix distribution. Multiphoton microscopy can extend imaging depth in thicker specimens while reducing phototoxicity and spinning-disk confocal systems may improve temporal resolution for dynamic imaging [167]. Time-lapse fluorescence microscopy is also valuable for monitoring biofilm development, dispersal, and antimicrobial responses in real time [168]. In addition, super-resolution techniques such as structured illumination microscopy (SIM) [169], stimulated emission depletion (STED) [170], and single-molecule localization methods including Photoactivated Localization Microscopy (PALM) [171] and Stochastic Optical Reconstruction Microscopy (STORM) [172] can provide sub-diffraction spatial detail, enabling finer analysis of matrix organization, cell–cell interactions, and subcellular localization of biofilm-associated molecules. When paired with quantitative image-analysis platforms such as COMSTAT or related software [173], these imaging datasets can be converted into robust structural descriptors, including biomass, average and maximum thickness, roughness, and porosity.

Several non-fluorescence microscopy techniques also contribute important structural information. Bright-field and phase-contrast microscopy are commonly used for routine observation of attached biomass and early-stage biofilm development [174], while differential interference contrast (DIC) microscopy improves contrast in unstained live samples and is useful for tracking surface colonization and microcolony formation [175]. In situ hybridization-based imaging, including fluorescence in situ hybridization (FISH) and peptide nucleic acid FISH, can be integrated with fluorescence microscopy or confocal platforms to identify the spatial organization of specific taxa within polymicrobial biofilms [176]. These approaches are especially valuable when species distribution or interspecies interactions are central to the study question.

For endpoint ultrastructural analysis, electron microscopy remains indispensable. Scanning electron microscopy (SEM) provides detailed views of biofilm surface architecture, cell morphology, and matrix-associated fibrillar material [177]. SEM can sometimes reduce dehydration artifacts by allowing imaging under more hydrated conditions. Transmission electron microscopy (TEM) reveals internal ultrastructure and fine details of cell envelopes and matrix-associated material at very high resolution [172,178]. Cryo-electron microscopy and cryo-electron tomography offer improved preservation of native ultrastructure by minimizing fixation and dehydration artifacts, although they are technically demanding and less commonly used for routine studies [179]. Together, these methods provide insights that are not achievable with conventional light microscopy, but they usually require extensive sample preparation and are low throughput. Atomic force microscopy (AFM) adds another dimension to biofilm analysis by generating nanoscale topographical maps and measuring biomechanical properties such as adhesion, stiffness, elasticity, and viscoelasticity [75,180]. Because biofilm behavior is strongly influenced by matrix mechanics, AFM is particularly useful for linking structure with physical function.

Chemical imaging methods can further complement structural microscopy by adding molecular information to spatial observations [171]. Raman microscopy, including confocal Raman microscopy and surface-enhanced Raman spectroscopy (SERS), enables label-free molecular characterization and spatial mapping of matrix components, metabolites, and other biomolecular signatures within biofilms [171,181,182,183]. Depending on the application, correlative and other spectroscopic imaging approaches can also be used to integrate morphology with chemical composition.

Overall, microscopy-based platforms provide a multiscale toolbox for studying biofilms, from bulk architecture to nanoscale structure and chemical organization, although many of the highest-resolution approaches remain specialized and relatively low throughput.

### 5.3. Real-Time and Label-Free Biosensors

Label-free optical and electrical biosensors are increasingly being developed for real-time monitoring of bacterial adhesion and early biofilm formation on relevant surfaces [184,185]. Fiber-optic interferometric and resonant platforms detect small changes in refractive index or optical path length associated with surface colonization and biomass accumulation [185,186]. Silicon nanowire field-effect transistor (SiNW-FET) platforms are attractive because they enable highly sensitive, real-time, label-free electrical detection and are compatible with miniaturized device formats [187,188]. In biofilm-focused applications, SiNW-FET systems have also been used for rapid, label-free monitoring of bacterial metabolic activity in microfluidic formats [189]. These technologies are promising, but clinical translation still depends on solving practical issues such as biofouling, calibration in complex biological fluids, and specificity in polymicrobial settings [184,185].

### 5.4. Molecular and Omics Approaches

Molecular techniques PCR, qPCR, and next-generation sequencing (NGS) including 16S rRNA amplicon sequencing, metagenomic sequencing can define species composition, identify genetic determinants (including antimicrobial-resistance genes) and infer functional potential [190]. Downstream activity layers (meta transcriptomics, meta proteomics, and metabolomics) capture in situ gene expression, protein production, and metabolite fluxes, enabling multi-omics frameworks to model biofilm behavior and metabolic exchange [191,192].

When integrated with imaging and in situ sensors, multi-omics approaches can connect spatial organization to gene expression patterns, enzyme activity, and metabolic information that is critical for designing therapies that target specific biofilm niches and their metabolic vulnerabilities [193,194,195]. High-throughput 16S rRNA gene sequencing, in particular, enables detailed bacterial typing (often to the species level) and can detect fastidious organisms or taxa that are frequently missed by culture-based methods [193,196,197]. By sequencing variable regions of the 16S rRNA gene and aligning reads to curated reference databases, investigators can reconstruct community structure, estimate relative abundances, and track changes in key pathogen or commensals over time or in response to a treatment [198]. Although taxonomic resolution may be limited for closely related species, results can be influenced by primer and database biases. 16S rRNA sequencing remains a foundational approach for characterizing the bacterial communities in complex clinical and environmental biofilms [199,200,201].

### 5.5. Practical Considerations for Clinical Monitoring

In clinical settings, biofilm detection must balance sensitivity, specificity, and timeliness to guide interventions while minimizing false positives that could lead to unnecessary treatments [202,203]. Routine culture of device explants, tip cultures (e.g., catheter tips), sonication followed by culture or molecular assays (like PCR), and imaging (e.g., SEM, confocal) remain core tools, but require careful sample handling to avoid contamination and ensure reproducibility [199]. For prosthetic infections, sonication of explanted hardware combined with culture and molecular testing increases diagnostic yield by dislodging adherent communities [202,204]. This thought demands specialized equipment and may not be feasible in resource-limited settings. Real-time sensor integration, such as NIR spectroscopy or metabolic-tracking biosensors for indwelling devices, represents a research frontier with strong translational potential for continuous, non-invasive monitoring, potentially reducing patient risks from repeated sampling [205]. Practical considerations include cost-effectiveness, operator training, integration with electronic health records, validation across diverse patient populations to address accuracy and equity and combining methods for comprehensive insights into biofilm persistence.

## 6. Clinical Significance of Biofilms in Diseases

Biofilms are central to the pathogenesis, persistence, and recurrence of many chronic and device-associated infections [206,207,208,209]. Their ecological properties translate into major clinical challenges, including impaired eradication, relapses, persistent inflammation, and progressive tissue injury [207,210].

In the respiratory tract, cystic fibrosis is the best-described example of a chronic biofilm infection, with persistent airway colonization by *P. aeruginosa* [41,211]. These infections often coexist with other organisms, including *S. aureus*, *Burkholderia cepacia* complex, and *Stenotrophomonas* spp., within polymicrobial airway communities [41,212,213]. In CF airways, mucoid and often alginate-rich *P. aeruginosa* biofilms develop within thick mucus, where oxygen and nutrient gradients promote metabolic heterogeneity and antibiotic tolerance [41,214]. Similar chronic biofilm-associated colonization occurs in non-cystic fibrosis bronchiectasis, in which *Haemophilus influenzae*, *Moraxella catarrhalis*, *S. aureus*, and *P. aeruginosa* are among the most common pathogens [212,215]. Biofilms are also implicated in chronic rhinosinusitis, where *S. aureus*, *P. aeruginosa*, and coagulase-negative staphylococci have been detected and linked to poorer outcomes [216,217,218]. In recurrent otitis media, biofilms formed by non-typeable *H. influenzae*, *Streptococcus pneumoniae*, and *M. catarrhalis* can persist on the middle-ear mucosa and contribute to recurrent disease [219,220].

Chronic wounds are another major clinical setting in which biofilms are important [39,221]. Diabetic foot ulcers (DFU), pressure injuries, and venous leg ulcers commonly harbor polymicrobial biofilms containing *S. aureus*, *P. aeruginosa*, anaerobes, and other Gram-negative organisms [39,221]. These communities sustain chronic inflammation, impair re-epithelialization, and decrease responsiveness to antimicrobial therapy [39,221]. As a result, management usually requires local wound care and debridement together with appropriate off-loading and antimicrobial therapy when indicated [221].

Bone and implant-associated infections are classic biofilm-mediated diseases [222,223]. Staphylococci predominate in osteomyelitis and periprosthetic joint infection, whereas *Cutibacterium acnes* is especially important in shoulder arthroplasty infection [25,209,224,225]. Once established on bone or implant surfaces, these infections are difficult to eradicate and commonly require surgical debridement, implant revision or exchange, and prolonged antimicrobial therapy [209,223].

Medical devices provide particularly favorable surfaces for biofilm establishment. Central venous catheters are major biofilm-prone devices, and common catheter-related bloodstream infection pathogens include coagulase-negative staphylococci, *S. aureus*, *Enterococcus* spp., Gram-negative bacilli, and *Candida* spp. [226]. Urinary catheters frequently develop biofilms, and *Proteus mirabilis* is especially important because crystalline biofilms cause encrustation and blockage [227,228,229]. Endotracheal-tube biofilms are a recognized risk factor for ventilator-associated pneumonia and commonly contain *Pseudomonas*, *Klebsiella*, *Staphylococcus*, and *Acinetobacter* spp. [230].

Biofilms also play a major role in oral disease [231,232]. Dental plaque is a complex multispecies biofilm, and dental caries reflects acidogenic and aciduric community shifts in which mutans streptococci remain important but are not the sole contributors [233]. In periodontitis, dysbiotic subgingival biofilms enriched in *Porphyromonas gingivalis*, *Treponema denticola*, and *Tannerella forsythia* are strongly associated with chronic inflammation and periodontal tissue destruction [232,234]. Related polymicrobial communities also colonize dental implants and are central to peri-implant mucositis and peri-implantitis [231,235,236].

Biofilms have also been implicated in complications around breast implants, particularly capsular contracture [237,238]. Skin-associated organisms, especially *S. epidermidis* and *C. acnes*, have repeatedly been detected on implant-associated capsules and biofilms [30,239,240]. These findings support a role for chronic low-grade bacterial colonization and inflammation, although any causal link between biofilms and systemic symptom syndromes remains under active investigation [241].

**Figure 3:**
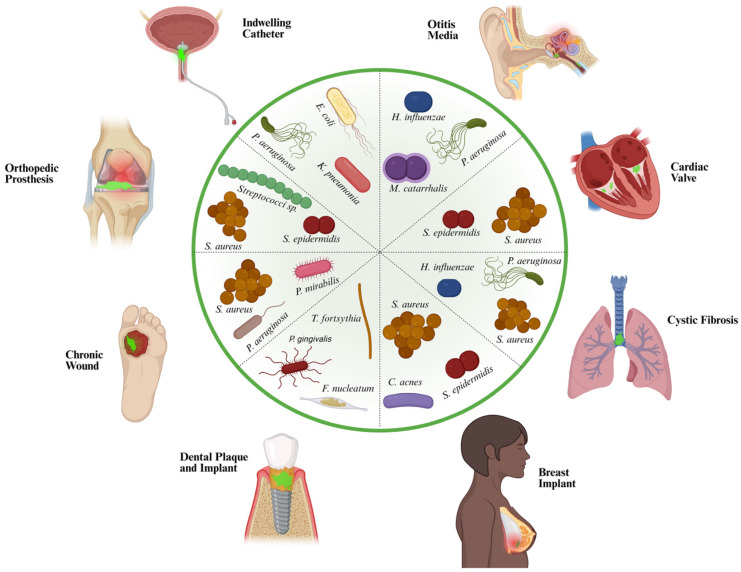
Human infections in which biofilms drive chronicity, recurrence, and treatment failure. Representative spectrum of biofilm-associated infections across tissues and indwelling medical devices. Examples include device-associated infections (e.g., intravascular/urinary catheters, prosthetic joints, cardiac valves and chronic tissue infections (e.g., cystic fibrosis, diabetic foot ulcers and breast implants) and surface biofilms (e.g., dental plaque/periodontitis, otitis media).

Across these diverse settings, common themes include multispecies organization, matrix-mediated protection, chemical microgradients, and enhanced tolerance to host defenses and antibiotics. These properties help explain why biofilm-associated infections are often chronic, relapsing, and difficult to eradicate. Persister cells are especially important because they can survive bactericidal therapy without a stable increase in MIC. After antibiotic concentrations fall, persisters can resume growth and repopulate the infection, contributing to relapses. Longitudinal studies in cystic fibrosis have shown the emergence of high-persister *P. aeruginosa* lineages during chronic airway infection. In a murine catheter-associated biofilm model, *S. aureus* biofilm cells showed reduced membrane potential and were associated with reduced clearance, as is consistent with persister enrichment [242]. Overall, the clinical implication is consistent across organ systems (Figure 3). Biofilm ecology promotes persistent infection, sustained immune activation, treatment failure, and recurrence, often necessitating combined mechanical, surgical, and pharmacological strategies with stronger emphasis on prevention and early detection [207,208].

## 7. Therapeutic Strategies

**Figure 4:**
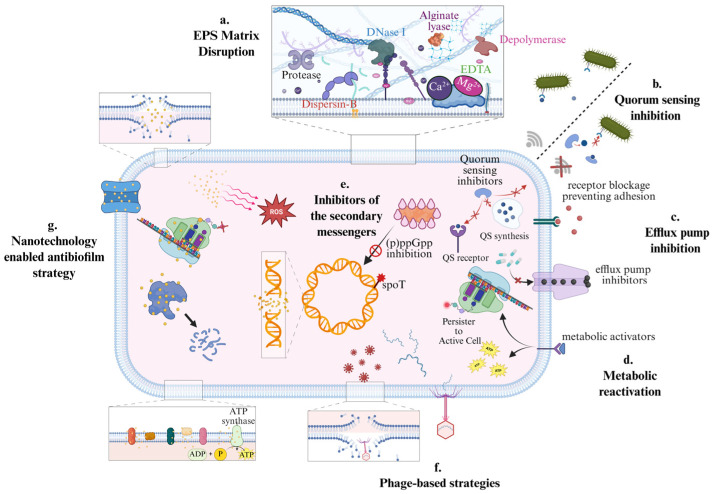
A layered strategy for prevention and treatment of biofilm infections. (**a**) EPS-targeting agents, including proteases, DNase I, depolymerases, alginate lyases, and EDTA, destabilize the matrix and enhance antibiotic penetration. (**b**) Quorum-sensing inhibitors disrupt signal synthesis, receptor binding, and intercellular communication, thereby reducing biofilm maintenance and virulence. (**c**) Efflux pump inhibitors prevent antibiotic extrusion and resensitize biofilm cells. (**d**) Metabolic activators enhance ribosomal activity and respiratory function, restoring susceptibility to antibiotic killing. (**e**) Inhibitors of the secondary messengers (p)ppGpp and cyclic di-AMP suppress stress-adaptive and survival pathways that support persistence. (**f**) Bacteriophages directly lyse bacterial cells. (**g**) Nanoparticles exert antimicrobial effects by disrupting membranes, impairing the electron transport chain, inactivating enzymes and ribosomes, and generating ROS that cause DNA damage.

Eradication of biofilm-associated infections involving catheters, implants, prosthetic joints, chronic wounds, CF airways, and dental surfaces is particularly challenging. In real-world clinical practice, effective management depends on combining source control with targeted antimicrobial treatment. Source control includes removal or exchange of infected devices when feasible, along with drainage of infected collections and surgical or mechanical debridement of affected tissues. This combined approach remains the backbone of therapy [243]. However, there is increasing use of biofilm-targeted and biofilm-disrupting adjunctive strategies in select clinical settings, and many of these approaches are currently being evaluated in clinical trials [244]. A practical framework for integrating these emerging therapies is a mechanism-guided, layered treatment design that addresses both the physical biofilm structure and the altered physiology of biofilm-associated organisms [245]. A framework for anti-biofilm therapy combining matrix disruption, targeted antimicrobials, and adjunct approaches is presented in Figure 4.

### 7.1. EPS Matrix Disruption

Matrix disruption is often a rational first pharmacologic step in biofilm-directed therapy because it enhances molecular diffusion and exposes embedded cells to both host immune defenses and antimicrobial agents. Enzymatic strategies are typically chosen to match the dominant chemical constituents of the extracellular matrix. For example, DNase targets extracellular DNA, a key structural component of many biofilms [246], alginate lyase degrades alginate-rich matrices characteristic of *P. aeruginosa* biofilms [247], and Dispersin B hydrolyzes poly-N-acetylglucosamine (PNAG) containing polysaccharides, promoting biofilm detachment [248,249]. Structural and biochemical studies further support the specificity and mechanisms of action of these enzymes [250].

In clinical and applied settings, matrix-disrupting agents are rarely used in isolation. Instead, they are commonly combined with physical interventions such as surgical debridement, device removal or exchange, and mechanical cleaning, all of which rapidly reduce total biofilm biomass and diminish the reservoir of persister cells, capable of reseeding infection. Importantly, weakening the biofilm matrix and reducing biomass create conditions in which subsequent antimicrobial therapy, the therapeutic “payload,” including antibiotics, bacteriophages, or immune effectors, can more effectively penetrate the biofilm and exert bactericidal activity. Thus, matrix disruption and biomass reduction function as enabling steps that increase the likelihood of success of downstream antimicrobial interventions. Matrix-disrupting strategies have been investigated against several clinically important biofilm-forming pathogens. Enzymatic approaches such as DNase I, alginate lyase, and Dispersin B have demonstrated biofilm-dispersing activity against organisms including *P. aeruginosa*, *S. aureus*, *S. epidermidis*, and *A. actinomycetemcomitans*. DNase I disrupts extracellular DNA scaffolding and reduces biofilm biomass in *P. aeruginosa* and *S. aureus* biofilms, typically producing a biofilm-disruptive rather than directly bactericidal effect. It markedly enhances antibiotic activity when combined with agents such as tobramycin or ciprofloxacin. Similarly, Dispersin B hydrolyzes poly-N-acetylglucosamine (PNAG) and has been shown to detach *S. epidermidis* and *A. actinomycetemcomitans* biofilms, resulting in increased susceptibility to antimicrobial agents. In most experimental systems, these enzymes primarily reduce matrix integrity and biomass, thereby enabling subsequent antimicrobial therapies to achieve bactericidal effects.

### 7.2. Phage-Based Therapeutics as Precision Anti-Biofilm Adjuncts

Phage-based strategies are appealing for biofilm control because they combine species or strain-level specificities with mechanisms capable of functioning within structured communities. Intact bacteriophages can replicate at the infection site and lyse susceptible bacteria, while phage-derived depolymerases can degrade polysaccharide components of biofilm matrix, thereby enhancing antimicrobial penetration and activity [251]. A clinically visible offshoot of the “phage enzyme” concept is the development of enzybiotics or lysins (phage-derived cell wall hydrolases) [252]. Despite strong mechanistic rationale and promising preclinical data, clinical translation is not assured. For example, a large phase 3 trial evaluating exebacase, an anti-*S. aureus* lysin, as an adjunct to standard-of-care antibiotics in *S. aureus* bacteremia, failed to demonstrate superiority on its primary efficacy endpoint. This outcome highlights the challenge of translating biofilm-active or highly targeted antimicrobial mechanisms into benefit across heterogeneous, invasive infection settings [253]. From an operational perspective, phage-based therapeutics continue to face practical barriers, including the need for precise host-range matching, challenges in manufacturing and regulatory standardization, and complex interactions with the host immune system. Nonetheless, phages and phage-derived enzymes remain credible adjunctive candidates, particularly when integrated into combination strategies that include matrix-disrupting agents and conventional antibiotics to overcome biofilm-associated tolerance. Phage-based anti-biofilm approaches have been most extensively studied in *S. aureus* (including MRSA) and *P. aeruginosa* biofilms. Lytic bacteriophages can penetrate structured biofilms and produce direct bactericidal effects through bacterial lysis, while phage-derived depolymerases degrade polysaccharide components of the extracellular matrix. Experimental studies have demonstrated significant reductions in viable cell counts and biofilm biomass in *P. aeruginosa*, *S. aureus*, and *A. baumannii* biofilms following phage treatment. In addition, synergistic activity has been observed when phages are combined with conventional antibiotics, leading to enhanced killing of biofilm-embedded cells. However, clinical translation remains challenging. For example, although early studies suggested promise for the anti-staphylococcal lysin exebacase, a large phase III trial in *S. aureus* bacteremia [254] did not demonstrate superiority over standard antibiotic therapy alone, highlighting the complexity of translating targeted biofilm therapies into heterogeneous clinical settings.

### 7.3. Local Delivery for Device-Associated Biofilms: Locks, Coatings, and Hydrogels

The most clinically actionable anti-biofilm innovations are often device-centered, as local delivery can achieve antimicrobial concentrations that exceed systemic levels by orders of magnitude. “Antimicrobial lock therapy” exemplifies this approach by targeting intraluminal catheter biofilms through prolonged exposure to high-concentration antibiotics. Taurolidine-containing lock solutions are supported by evidence from prevention-focused clinical studies and meta-analyses [255]. Biofilm-eradicating lock formulations extend this concept by combining agents that disrupt the extracellular matrix with antimicrobials that kill embedded cells. An “optimal” lock strategy has been shown in experimental studies to rapidly eradicate organisms within established biofilms [256], and successful clinical catheter salvage using a minocycline–EDTA–25% ethanol lock solution has been reported in patients with catheter-related bloodstream infections when catheter removal is not feasible [257].

Beyond intravascular devices, anti-biofilm strategies have also been developed for orthopedic and trauma implants. Fast resorbable antibiotic-loaded hydrogel coatings (DAC^®^) have undergone clinical evaluation, including a multicenter randomized trial in internal osteosynthesis [258] and an early joint arthroplasty study demonstrating reduced rates of early surgical site infections without apparent safety concerns [259]. In addition to lock solutions and hydrogel coatings, several advanced delivery systems are being developed to improve antimicrobial penetration into biofilm matrices. Nanoparticle-based carriers, including liposomes, polymeric nanoparticles, and metallic nanoparticles (e.g., silver or chitosan-based systems), can enhance drug diffusion through the EPS by protecting antimicrobial agents from inactivation and facilitating sustained local release. Liposomal formulations have been particularly investigated for delivering antibiotics such as aminoglycosides directly into biofilm structures [260]. Stimuli-responsive delivery systems, including pH-sensitive or enzyme-responsive hydrogels, are also being explored to trigger localized drug release in the acidic or enzyme-rich microenvironments characteristic of mature biofilms. In parallel, antimicrobial peptide-loaded coatings and enzyme-functionalized materials capable of degrading extracellular matrix components (e.g., DNase or dispersin B) have shown promise in improving antibiotic access to embedded cells. Collectively, these diverse formulations illustrate the growing versatility of local delivery technologies aimed at overcoming biofilm-associated diffusion barriers and enhancing therapeutic efficacy.

### 7.4. Quorum Sensing Inhibition and Anti-Virulence “Disarmament” Strategies

Quorum-sensing inhibitors (QSIs) and related anti-biofilm small molecules target coordination, not viability, disarming collective behaviors such as maturation, matrix production, and virulence expression [261]. This “disarm rather than kill” framing is clinically appealing because it could, in principle, reduce resistance selection while sensitizing communities to immunity and antibiotics. A closely related, well-studied chemical class is 2-aminoimidazole–based compounds, which can inhibit biofilm development and interact with response regulators that control biofilm programs. In *A. baumannii*, the response regulator BfmR (a master controller of biofilm development) was identified as a target for a 2-AI anti-biofilm agent, providing a mechanistic anchor for why these molecules can disrupt community behaviors [262]. In practice, QSIs/anti-biofilm modulators are best conceptualized as adjunct layers. They are unlikely to clear established infections alone, but they may reduce relapses and improve antibiotic susceptibility when paired with matrix disruption and adequate drug delivery.

Quorum-sensing inhibition has been investigated particularly in *P. aeruginosa*, *A. baumannii*, and *S. aureus* biofilms. Small-molecule inhibitors such as 2-aminoimidazole derivatives disrupt regulatory pathways controlling biofilm maturation and virulence gene expression [263]. In experimental models of *A. baumannii*, inhibition of the response regulator BfmR significantly reduced biofilm formation and increased susceptibility to antimicrobial agents [264]. Unlike traditional antibiotics, these agents generally exhibit anti-biofilm or bacteriostatic-like effects rather than direct bactericidal activity, functioning primarily as sensitizing adjuncts that improve antibiotic penetration or host immune clearance.

### 7.5. Targeting Persister Cells Through Metabolic Reactivation

Persisters are not genetically resistant; they are phenotypically tolerant, often because dormancy reduces antibiotic target engagement and active uptake. A practical anti-persister concept is metabolic reactivation, transiently pushing dormant cells into a state where bactericidal drugs work. A landmark example showed that certain metabolites can potentiate aminoglycoside killing by enabling uptake and restoring lethal activity against otherwise tolerant persisters [265]. Translationally, this motivates combination logic: deliver a bactericidal antibiotic (or local high concentration) while simultaneously providing a metabolic “wake-up” signal, ideally in a controlled, localized manner to limit off-target effects. In biofilm therapy design, persister targeting is the last resort. It matters most after you have already reduced biomass and improved penetration (via debridement and/or matrix disruption). Otherwise, persisters simply remain protected behind diffusion barriers, and reactivation signals may not reach them uniformly across the biofilm’s chemical gradients. Persister targeting should be viewed more broadly than a simple “wake-up” strategy. In biofilms, persisters emerge from stochastic phenotypic switching, nutrient and oxygen limitation, and low membrane potential. Stress-responsive signaling networks such as the stringent response, while quorum sensing, c-di-GMP pathways, and biofilm-specific regulators including SagS and BrlR can further stabilize tolerant states and reduce intracellular antibiotic accumulation [266]. Metabolic reactivation nevertheless remains a rational anti-persister approach because transient restoration of respiration or proton motive force can resensitize dormant cells to antibiotics that depend on active uptake or active targets. A landmark example showed that selected metabolites can potentiate aminoglycoside killing of persisters by restoring uptake and lethal activity. Translationally, this supports layered combination therapy: first reduce biomass and diffusion barriers, second deliver an effective antimicrobial payload, and third to pair that payload with a tolerance-disrupting adjuvant such as metabolic stimulation or stress-response/efflux modulation. Without the first two steps, dormant subpopulations remain shielded within heterogeneous microenvironments and are unlikely to be reached uniformly across the biofilm. For this reason, localized or device-directed delivery may be attractive for future anti-persister therapy regimens.

Metabolic reactivation strategies have been studied primarily in *P. aeruginosa*, *E. coli*, and *S. aureus* biofilms. Certain metabolites, including fumarate, mannitol, and glucose, can stimulate cellular respiration and restore proton motive force, thereby facilitating aminoglycoside uptake and restoring bactericidal activity against persister populations. Experimental studies in *P. aeruginosa* biofilms have demonstrated that metabolic stimulation combined with aminoglycosides significantly increases killing of otherwise tolerant cells [265]. These findings support the concept that persister-targeting therapies function primarily as adjuvant approaches that convert dormant populations into antibiotic-susceptible states.

### 7.6. Efflux Pump Inhibition and Suppression of Biofilm Stress Responses

Biofilm-associated tolerance is driven in part by regulatory adaptations, as biofilm communities activate transcriptional programs that reduce intracellular antimicrobial exposure and enhance survival under stress. In *P. aeruginosa*, the MerR-like transcriptional regulator BrlR plays a central role in mediating high-level antimicrobial tolerance in biofilms by linking biofilm growth to multidrug tolerance pathways [267]. This mechanistic insight supports two complementary adjunctive strategies, inhibition of efflux pumps to increase intracellular drug accumulation, and targeting of stress-response pathways to attenuate community-wide tolerance. Clinically, translation of these approaches remains challenging. Selective and safe inhibitors are limited, and broad efflux pump inhibition carries risks of toxicity and adverse drug interactions. Consequently, tolerance-targeting strategies are best viewed as enabling components rather than stand-alone therapies. Mechanistically, BrlR illustrates why bacteria that appear susceptible in standard laboratory tests can persist during infection [267]. Within a biofilm, cells activate regulatory programs that protect them from antibiotics, so their survival is determined more by this coordinated tolerance response than by the minimum inhibitory concentration. When incorporated into layered treatment regimens that combine matrix disruption, antimicrobial therapy, and tolerance-modulating agents, this approach aims to reduce post-treatment rebound and prevent recolonization.

### 7.7. Nanotechnology-Enabled Delivery and Multi-Agent Packaging

Nanotechnology-enabled antibiofilm delivery tackles two core barriers: poor penetration through the EPS and the need for sustained, high local antimicrobial levels. Liposomes, nanogels and polymeric nanoparticles can be engineered with “biofilm-affinity” surfaces (e.g., charge-switching coatings) so they bind anionic matrix components, move deeper into the biofilm, and then release the drug where bacteria reside [268]. In one clear example, layer-by-layer nanoparticles that convert from net negative to net positive under the lower-pH conditions typical of *P. aeruginosa* biofilms penetrated more effectively and, when loaded with tobramycin, produced greater CFU reductions than free drug in a hyper-biofilm mutant [268]. Layered therapy can also be built into one carrier. DNase I functionalized ciprofloxacin nanoparticles degraded extracellular DNA while providing controlled antibiotic release, markedly reducing established *P. aeruginosa* biofilm biomass and viability in a flow-cell model [269]. Multi-agent payloads are feasible too; liposomal co-delivery of ciprofloxacin plus the quorum-sensing molecule, farnesol, increased depth of killing and lowered the ciprofloxacin concentration needed for comparable biofilm inhibition [270]. More broadly, biofilm-responsive factors (pH, enzymes, ROS/redox cues) and “sense-and-release” theranostics are being developed to make delivery context-aware in chronic wounds and airway disease [271,272]. Nanoparticle-based anti-biofilm systems have been investigated against several clinically relevant pathogens including *P. aeruginosa*, *S. aureus*, *S. epidermidis*, and *E. coli*. Silver nanoparticles and nitric oxide-releasing nanomaterials have demonstrated direct bactericidal effects through membrane disruption, oxidative stress generation, and interference with cellular respiration [273]. In contrast, polymeric nanoparticles and liposomal systems often function primarily as drug delivery enhancers, improving antibiotic penetration into biofilms and sustaining local drug concentrations. Experimental studies using charge-switching nanoparticles loaded with tobramycin have demonstrated enhanced killing of *P. aeruginosa* biofilms, while DNase-functionalized nanoparticles have reduced biofilm biomass and viability in flow-cell models [274].

### 7.8. Energy-Based and Non-Drug Modalities for Accessible Biofilms

For superficial or accessible biofilms (chronic wounds, dental plaque, some device-adjacent infections), energy-based modalities provide nontraditional killing/disruption that does not rely on systemic drug penetration. Cold atmospheric plasma (CAP) generates reactive species and has been evaluated clinically; in a randomized trial for diabetic foot ulcers, adjunctive CAP improved wound-healing metrics compared with placebo/standard care [275]. Antimicrobial photodynamic therapy (aPDT) uses a photosensitizer plus visible light to generate reactive oxygen species that damage microbes and can reduce dental biofilm burden; randomized clinical trial evidence exists in pediatric dental biofilm contexts [276]. “Electroceutical” dressings create low electric fields in moist environments and are designed to suppress biofilms while supporting healing; a prospective randomized controlled study evaluated a fabric-based wireless electroceutical dressing against acute trauma/burn wound biofilm infection [277]. Practically, these tools fit the layered model as front-end disruptors that reduce biomass and weaken biofilm defenses before (or alongside) topical/systemic antimicrobials. Energy-based approaches have been evaluated primarily in polymicrobial wound biofilms, *S. aureus* biofilms, and oral plaque biofilms dominated by Streptococcus species. Cold atmospheric plasma generates reactive oxygen and nitrogen species capable of producing rapid bactericidal effects on exposed biofilm surfaces. Similarly, antimicrobial photodynamic therapy has demonstrated substantial reductions in *S. mutans* and mixed oral biofilms through light-activated generation of reactive oxygen species. Clinical trials in chronic wound settings have shown improved microbial clearance and wound healing outcomes when these modalities are used as adjunctive therapies [278].

### 7.9. Synthetic Small Molecules, Natural Products, and Antimicrobial Peptides

A broad spectrum of synthetic small molecules and natural compounds can interfere with biofilm formation by preventing adhesion, disrupting matrix assembly, or inhibiting quorum sensing, with examples often including octyl gallate and plant-derived polyphenols [279]. Their appeal is that some may reduce biofilm formation without strong growth inhibition, potentially lowering classic resistance selection, though potency, pharmacokinetics, and safety must be carefully validated. Beyond “anti-adhesion,” several translational strategies target biofilm physiology and dispersal. In cystic fibrosis relevant contexts, OligoG (alginate oligosaccharide) has been described as disrupting biofilms and potentiating antimicrobials as an inhaled approach [280]. Nitric oxide (NO) is another dispersal-linked molecule: NO can be antimicrobial and can trigger dispersal pathways at low concentrations; device concepts include NO-releasing urinary catheters aimed at preventing colonization and early biofilm formation [281]. Gallium, acting as an iron mimetic, disrupts iron-dependent processes important for chronic *Pseudomonas* physiology. Inhaled gallium citrate (e.g., AR 501) and intravenous gallium approaches have been explored in cystic fibrosis-related infection settings [282].

Antimicrobial peptides (AMPs) and peptide-like agents are attractive because many are rapidly bactericidal and can act under conditions where conventional antibiotics struggle. Beyond membrane disruption, many AMPs also interfere with intracellular pathways that regulate biofilm development. Several AMPs have been shown to penetrate bacterial cells and interact with intracellular targets, including nucleic acids, ribosomes, and key metabolic enzymes [283]. By binding DNA or RNA, some peptides inhibit transcriptional programs required for adhesion, matrix production, and stress adaptation during early biofilm formation. Others interfere with protein synthesis or disrupt ATP generation, thereby limiting the metabolic capacity needed for biofilm maturation. Importantly, certain AMPs also modulate quorum-sensing networks and intracellular second-messenger pathways such as cyclic-di-GMP signaling, which plays a central role in biofilm formation and dispersal. Through these combined actions, membrane permeabilization, intracellular targeting, and interference with regulatory signaling, AMPs can suppress biofilm initiation, destabilize established communities, and enhance the susceptibility of embedded bacteria to conventional antibiotics. However, systemic translation has been slower than topical development due to stability and toxicity constraints. Omiganan (a synthetic cationic peptide) illustrates the topical high concentration rationale relevant to device exits and skin interfaces, in vitro activity against pathogens implicated in catheter-associated infections, including fungal pathogens [284] and contemporary bacterial pathogens commonly responsible for catheter-associated infections [285], has been documented. From a biofilm-therapy design standpoint, peptides often occupy the “payload” layer, especially when delivered topically or locally. Other adjuncts (matrix enzymes, coatings, or physical removal) improve access to the embedded community. Natural products and synthetic non-growth inhibitory compounds are frequently discussed as anti-biofilm adjuvants, but potency, pharmacokinetics, and safety remain the key hurdles before routine clinical use [286,287]. Antimicrobial peptides and related synthetic molecules have demonstrated antibiofilm activity against a range of pathogens including *S. aureus*, *P. aeruginosa*, and *E. faecalis*. Many peptides exert direct bactericidal activity through membrane permeabilization, while others interfere with intracellular processes such as transcription, translation, and quorum-sensing signaling. The human cathelicidin LL-37, for example, has been shown to produce substantial reductions in *S. aureus* biofilm viability, while synthetic peptides such as omiganan exhibit activity against pathogens associated with catheter-related infections [288]. Although these agents show strong in vitro antibiofilm activity, systemic clinical use remains limited due to concerns regarding peptide stability, toxicity, and pharmacokinetics.

### 7.10. Immune Targeting and Antimicrobial Peptides: Promise with Mixed Clinical Translation

Anti-virulence immunotherapies aim to neutralize pathogenesis and bacterial adhesion rather than directly kill bacteria, with the theoretical advantage of reducing selection pressure for resistance. A well-known clinical example is MEDI3902 (Gremubamab), a bispecific monoclonal antibody targeting *P. aeruginosa* PcrV (type III secretion) and Psl (a biofilm-associated exopolysaccharide). In a phase 2 trial, *P. aeruginosa* colonized ICU patients; MEDI3902 did not reduce the incidence of *Pseudomonas* nosocomial pneumonia compared with the placebo [289]. This outcome does not invalidate the immunotherapy approach but highlights key biofilm-associated infection realities. Progression from colonization to disease is multifactorial, and virulence pathways are often redundant. Clinical benefit may depend on careful patient selection as well as combination with optimized antimicrobial therapy and source-control measures. Vaccine development against biofilm-forming pathogens remains active, although no broadly adopted biofilm-targeted vaccine has yet become standard in major clinical indications.

The most promising therapeutic strategies are increasingly based on layered or sequential design. First, disrupting the EPS matrix through enzymes, depolymerases, ultrasound, or mechanical removal can reduce structural protection and improve diffusion of therapeutics. Followed by delivery of a concentrated antimicrobial payload, such as localized antibiotics, nanocarriers, or bacteriophages, to achieve effective activity within the biofilm. Finally, to disable communal resilience traits, including quorum-sensing inhibition, metabolic reactivation, or efflux inhibition to prevent recolonization, reduce persistence and limit the emergence or spread of antimicrobial resistance. Advances in multi-omics (metagenomics, metatranscriptomics, metabolomics), single-cell analytics, and computational modeling will enable precision targeting of biofilm vulnerabilities, including identification of persister-enriched niches, matrix composition, and key metabolic interactions sustaining dormancy. In this context, personalized strategies such as matching enzymatic adjuncts to dominant matrix chemistries, selecting phage cocktails according to isolate susceptibility, or tuning nanocarrier release to local pH conditions represent promising translational directions. The pathogen spectrum, biological effects (bactericidal, bacteriostatic, or biofilm-disruptive), and clinical applications of these emerging anti-biofilm strategies are summarized in Table 2.

## 8. Clinical Implications and Future Directions

The evidence synthesized in this review indicates that effective management of biofilm-associated infections is limited when these conditions are interpreted solely within a planktonic framework. Standard in vitro susceptibility testing, short courses of monotherapy, and a narrow focus on organism identity and MIC values often fail to capture the biological complexity of biofilm infections. Biofilms create protected microenvironments characterized by physiological heterogeneity, dormant or slow-growing subpopulations, and the frequent coexistence of multiple species or strains. These features generate a multilayered tolerance phenotype that cannot be fully explained by conventional antimicrobial resistance metrics.

In clinical practice, successful management of biofilm-associated infections therefore requires multimodal and layered strategies. While antimicrobial selection and dosing remain critical, they must be integrated with device management, surgical source control, and optimization of host factors such as perfusion, glycemic control, and immune competence. Infections involving prosthetic joints, cardiac devices, vascular grafts, or orthopedic hardware frequently cannot be eradicated with antibiotics alone. Decisions regarding device retention versus removal, surgical debridement strategies, and reconstruction are often central determinants of outcome. Similarly, in chronic wound infections, local measures such as debridement, drainage, and topical therapies are essential for reducing biofilm burden.

Emerging diagnostic approaches may help bridge the gap between the complex biology of biofilms and the tools currently available in clinical practice. Advanced imaging techniques, molecular diagnostics, and label-free detection methods offer the possibility of earlier recognition of biofilm involvement, improved risk stratification, and more accurate monitoring of treatment response. Point-of-care tests capable of detecting biofilm-associated molecular signatures, or imaging modalities that directly visualize biofilm structures on tissues or devices, could help clinicians determine when aggressive intervention is required and when conservative management is sufficient. However, successful integration of these technologies into clinical workflows will require rigorous validation, standardized interpretation frameworks, and clinician education.

Mechanistic insights into biofilm biology also point toward new therapeutic opportunities. Multi-omics studies and advanced modeling approaches have begun to identify context-specific vulnerabilities within biofilm communities, including dependencies on specific metabolic pathways, matrix components, and intercellular signaling networks. Combination therapies that pair conventional antibiotics with matrix-disrupting agents such as DNases, enzymes, or surfactants may improve antimicrobial penetration and immune access. Additional approaches, including bacteriophages, phage-derived enzymes, nanoparticles, targeted delivery systems, and metabolic adjuvants, offer strategies to concentrate on antimicrobial activity at the site of infection or sensitize dormant bacterial subpopulations that otherwise survive therapy.

These innovations must be developed within robust antimicrobial stewardship and regulatory frameworks. Layered treatment regimens combining biologics, enzymes, antimicrobial agents, and medical devices will require new regulatory pathways and clinical trial designs that reflect the chronic and device-associated nature of many biofilm infections. Stewardship programs will also need to consider not only antimicrobial selection and duration but also the sequencing and ecological consequences of complex therapeutic combinations.

Prevention represents a particularly important and often underutilized pillar of biofilm management. Clinically, this includes careful device selection, adherence to insertion and maintenance bundles, timely removal of unnecessary or infected devices, and early intervention when colonization is suspected. At the technological level, advances in biomaterials, surface engineering, and device architecture are being explored to reduce microbial attachment and biofilm formation. Incorporating a biofilm-aware perspective into infection prevention guidelines, device design, and quality improvement initiatives could substantially reduce the burden of biofilm-associated complications.

Finally, translating biofilm-centered insights into clinical practice will require sustained interdisciplinary collaboration among microbiologists, infectious disease specialists, surgeons, engineers, materials scientists, and regulatory authorities. The development of realistic preclinical models, standardized definitions of biofilm involvement, and harmonized clinical outcome measures will be essential for moving the field from conceptual advances to evidence-based clinical recommendations.

## 9. Conclusions

Bacterial biofilms are not simply an alternative mode of microbial growth but a clinically transformative state that reshapes how infections begin, persist, and respond to therapy. Across the conditions discussed in this review, a consistent theme emerges, once bacteria enter the biofilm state, infection can no longer be understood adequately through a purely planktonic framework. The extracellular polymeric substance matrix, coupled with spatial organization, metabolic stratification, and biofilm-specific transcriptional programs, creates communities that are structurally protected, physiologically heterogeneous, and far less susceptible to antimicrobial killing than standard laboratory testing would predict. This helps explain why infections involving devices, chronic wounds, airway secretions, implants, and other vulnerable niches so often show discordance between in vitro susceptibility and clinical response. Biofilm infections may involve isolates that are formally susceptible by MIC criteria yet remain persistent because embedded cells experience restricted drug penetration, altered pH and redox conditions, reduced metabolic activity, active efflux, stress-adapted survival pathways, and the presence of persister subpopulations capable of surviving prolonged antibiotic exposure. In parallel, biofilms impair effective immune clearance by shielding bacteria from phagocytosis, blunting complement and antimicrobial peptide access, and sustaining a state of ineffective but tissue-damaging inflammation. Together, these properties make biofilms a central biological explanation for chronic infection, relapses after apparently appropriate treatment, and the frequent need for repeated intervention.

A second major conclusion is that biofilm-associated infection must be approached as a microenvironmental and host-contextual problem rather than solely as a question of organism identity and antibiotic selection. This review highlights that biofilm formation and persistence depend not only on microbial factors but also on local environmental conditions and failures of host defense. Foreign-body surfaces, impaired perfusion, tissue necrosis, hyperglycemia, chronic inflammation, immune dysfunction, and compromised barrier integrity all create microenvironments in which biofilms can establish and endure. This is why successful management so often requires more than escalation of antimicrobial therapy. Source control, biomass reduction, and restoration of the local tissue environment are not ancillary measures but core therapeutic principles. Device removal or exchange, debridement, drainage, mechanical cleaning, and optimization of host factors such as perfusion and glycemic control may determine outcome as much as the antibiotic regimen itself. The same logic applies to emerging anti-biofilm strategies. Matrix-disrupting enzymes, bacteriophages, targeted delivery systems, metabolic adjuvants, and host-directed therapies are promising not because they replace conventional antimicrobials, but because they address the features of biofilm biology that antibiotics alone often cannot overcome. Likewise, improved diagnostics including advanced microscopy, molecular tools, and label-free detection methods will be valuable only insofar as they help clinicians recognize biofilm involvement earlier, define risk more accurately, and match interventions to the biological realities of the infected niche. A biofilm-aware clinical approach therefore requires integration of microbiology, surgery, device management, wound care, pharmacology, and host optimization together rather than in isolation.

Ultimately, the most important conclusion of this review is that biofilms should be recognized as a unifying principle in many of the most difficult infections encountered in modern medicine. They provide a mechanistic link between persistent colonization, chronic inflammation, treatment tolerance, and recurrent disease across diverse anatomical sites and clinical syndromes. Appreciating this continuity has practical implications for research, stewardship, prevention, and guideline development. Future progress will depend on moving beyond simplified models of acute planktonic infection toward more realistic definitions, diagnostics, and treatment paradigms that reflect the structured, adaptive, and relapsing nature of diseases involving biofilm. It will also require interdisciplinary collaboration among microbiologists, infectious disease specialists, surgeons, wound-care teams, engineers, and regulatory bodies to translate mechanistic insight into clinically validated strategies. Just as importantly, prevention must remain central. Use of improved biomaterials, careful device stewardship, timely removal of unnecessary foreign bodies, and early local intervention may prevent biofilms from becoming established in the first place. In this sense, the clinical significance of biofilms lies not only in their ability to explain therapeutic failure but also in their ability to redirect clinical thinking toward more rational, multimodal, and preventive care. Incorporating biofilm biology into routine clinical reasoning will be essential if persistent and recalcitrant infections are managed with greater precision, predictability, and long-term success.

## Figures and Tables

**Table 1:** Detection Methods for Biofilms in Clinical Settings.

Method	Key Techniques	Clinical Contexts	Advantages/Limitations	References
Fluorescence Imaging (e.g., MolecuLight)	Violet light excitation of bacterial porphyrins for real-time visualization of bacterial load/biofilm at bedside.	Chronic wounds, diabetic foot ulcers	High sensitivity, non-invasive, point-of-care; alerts to biofilm regions. Limited species specificity may overestimate the biofilm abundance in some cases.	Mayer et al., 2024 (PMID: # 39410520) [146]
Dual-Staining Microscopy (e.g., Maneval’s + Congo Red)	Light microscopy with dual stains to differentiate polysaccharide matrix (blue) from cells (magenta-red).	General clinical isolates, wounds, devices	Low-cost, accurate alternative to advanced imaging; matches microtiter assays. Not real-time or bedside for large areas.	Nirmala et al., 2024 (# 39587230) [147].
Phenotypic Biofilm Assays (TCP, CRA, Tube Method)	Tissue culture plate (most sensitive), Congo red agar, tube method for isolate confirmation from clinical samples.	Uropathogens, *S. aureus*, catheter isolates	Simple, low-cost for lab confirmation; correlates with resistance. In vitro only, not direct in vivo detection.	Khaddam et al., 2025 (# 41249920) [148]; Harika et al., 2020 (# 33343163) [149].
Molecular/Omics (16S NGS, Metagenomics, RNA-seq)	Shotgun metagenomics or full-length 16S from samples (e.g., wound swabs, peri-implant).	Chronic wounds, peri-implantitis, oral/CF biofilms	High-resolution taxonomic/functional profiling; detects polymicrobial shifts. Lab-based, time-consuming, expensive.	Xiao et al., 2022 (# 36389056) [150]; Joshi et al., 2025 (# 40858628) [151]; Dame-Teixeira et al., 2024 (# 39107803) [152]; Khan et al., 2023 (# 38032740) [30]
pH-FISH/Confocal Ratiometry	Confocal pH ratiometry + FISH for mapping local pH and taxa in intact biofilms.	Dental/oral biofilms, potential wound extension	Correlates taxa with microenvironments (e.g., acid-tolerant Streptococci). Lab-intensive; not bedside.	Del Rey et al., 2024 (# 39707459) [153]
Sonication + Culture/Molecular	Sonication of explants/hardware to dislodge biofilms, followed by culture/PCR/NGS.	PJI, orthopedic implants, catheters	Increases yield for adherent/fastidious organisms. Requires explantation; specialized equipment.	Khaddam et al., 2025 (# 41249920) [148]; Mikziński et al., 2024 (# 38930580) [154].

**Table 2:** Treatment/Therapeutic Strategies for Biofilms in Clinical Settings.

Therapeutic Strategy	Key Mechanisms	Representative Pathogens/Biological Effect	Clinical Contexts	Advantages/Limitations	References
Multimodal/Layered	Debridement/device removal, targeted antibiotics, matrix disruptors.	*S. aureus*, *S. epidermidis*, *P. aeruginosa*, *Enterococcus* spp.—Bactericidal (antibiotic dependent)	Prosthetic joint infection, chronic wounds, device infections	Current clinical standard; removes biomass and infection source/Invasive; recurrence possible if device retained	Haval et al., 2025 (# 41009844) [172]; Grari et al., 2025 (# 40261031) [290].
Matrix Disruption (Enzymes/Depolymerases)	DNase, alginate lyase, dispersin B, or phage-derived to degrade EPS.	*P. aeruginosa*, *S. aureus*, *S. epidermidis*, *Aggregatibacter actinomycetemcomitans*.—Biofilm-disruptive (adjunctive)	CF airways, chronic wounds, oral biofilms	Enhances antibiotic penetration and immune access/Limited efficacy as monotherapy	Wang et al., 2024 (# 38402083) [291].
Bacteriophage Therapy	Lytic phages, phage cocktails, lysins, depolymerases	*S. aureus* (MRSA), *P. aeruginosa*, *Acinetobacter baumannii.*—Bactericidal	MDR infections, chronic wounds, CF airway infection, prosthetic infections	High specificity; synergizes with antibiotics/Host-range constraints; regulatory barriers	Poniatovsky et al., 2025 (# 41229989) [292]
Nanotechnology-based delivery	Liposomes, polymeric nanoparticles, silver nanoparticles, charge-switching carriers	*P. aeruginosa*, *S. aureus*, *S. epidermidis*, *E. coli.*—Bactericidal or drug-delivery enhancement	Chronic wounds, implant infections, airway infections	Improved drug penetration; sustained release/Safety and translational challenges.	Asalipisheh et al., 2025 (# 41068623) [293]
Quorum Sensing Inhibition	Signal synthesis inhibitors, receptor blockers, 2-aminoimidazoles.	*P. aeruginosa*, *A. baumannii*, *S. aureus.*—Anti-virulence/bacteriostatic-like	Chronic airway and wound infections	Reduces virulence and biofilm maturation/Mostly preclinical.	Jiang et al., 2023 (# 37738984) [294]; Oliveira et al., 2023 (# 37054672) [295].
Local delivery systems	Antimicrobial lock therapy, taurolidine locks, antibiotic hydrogels, coated implants	*S. aureus*, *S. epidermidis*, Gram-negative catheter pathogens, *Candida* spp.—Bactericidal (high local concentration)	Catheters, orthopedic implants	Very high local antimicrobial levels/Limited to accessible devices	Lordelo et al., 2024 (# 38711646) [296].
Anti-persister metabolic activation	Metabolite supplementation, respiration stimulation, aminoglycoside potentiation	*P. aeruginosa*, *E. coli*, *S. aureus.*—Restores bactericidal antibiotic activity	Chronic biofilm infections, CF	Targets antibiotic tolerance mechanisms/Requires combination therapy	Marcel et al., 2014 (# 25374846) [297]
Antimicrobial peptides (AMPs)	Membrane disruption, quorum sensing interference, intracellular targeting	*S. aureus*, *P. aeruginosa*, *Enterococcus* spp.—Bactericidal	Wound infections, catheter exit sites	Rapid killing; broad antibiofilm activity/Stability and toxicity concerns	Cesar et al., 2025 (# 39530703) [298]
Energy-Based/Non-Drug	Cold atmospheric plasma, photodynamic therapy, electroceutical dressings	Polymicrobial wound biofilms, *S. aureus*, *S. mutans*. Surface bactericidal	Chronic wounds, dental biofilms	Antibiotic-independent Physical disruption/Limited tissue penetration.	Liu et al., 2025 (# 40050838) [299]
Immunotherapeutic strategies	Anti-virulence antibodies (e.g., MEDI3902), vaccine approaches	*P. aeruginosa*, *S. aureus*.—Anti-virulence/adjunctive	ICU infections, pneumonia prevention	Pathogen-specific targeting	Mixed clinical trial results [289]

## Data Availability

No new data were created or analyzed in this study. Data sharing is not applicable to this article.

## Abbreviations

AFM	Atomic Force Microscopy
aPDT	Antimicrobial Photodynamic Therapy
AMR	Antimicrobial Resistance
AMPs	Antimicrobial Peptides
BFI	Biofilm formation index
BII	Breast Implant Illness
c-di-GMP	Cyclic diguanylate monophosphate
CAP	Cold Atmospheric Plasma
CDC	Center for Disease Control and Prevention
CF	Cystic Fibrosis
CFU	Colony Forming Unit
CLABSI	Central line-associated bloodstream infection
CLSM	Confocal Laser Scanning Microscopy
CRA	Congo red agar
CT	Computed tomography
DAPI	4′,6-diamidino-2-phenylindole
DNA	Deoxyribonucleic acid
eDNA	Extracellular DNA
EPS	Extracellular Polymeric Substance
FISH	Fluorescence In Situ Hybridization
HGT	Horizontal Gene Transfer
ICU	Intensive Care Unit
MBEC	Minimum Biofilm Eradication Concentration
MBIC	Minimum Biofilm Inhibitory Concentration
MIC	Minimum Inhibitory Concentration
NETs	Neutrophil Extracellular Traps
NGS	Next Generation Sequencing
NIR	Near-Infrared
NO	Nitric Oxide
OMVs	Outer Membrane Vesicles
PALM	Photoactivated Localization Microscopy
PCR	Polymerase Chain Reaction
PDT	Photodynamic therapy
PIA	Polysaccharide intercellular adhesin
PMF	Proton Motive Force
PNAG	Poly-β-1,6-N-acetylglucosamine
PJI	Prosthetic Joint Infection
qPCR	Quantitative Real-Time PCR
QS	Quorum Sensing
QSI	Quorum Sensing Inhibitor
RNA	Ribonucleic acid
ROS	Reactive Oxygen Species
SERS	Surface-enhanced Raman spectroscopy
SEM	Scanning Electron Microscopy
SiNW-FETs	Silicon Nanowire Field Effect Transistors
SIM	Structured illumination microscopy
SOS	SOS DNA Damage Response System
SSBI	Systemic Symptoms Associated with Breast Implants
STED	Stimulated emission depletion
STORM	Stochastic Optical Reconstruction Microscopy
TCP	Tissue Culture Plate (biofilm assay)
TEM	Transmission Electron Microscopy
WHO	World Health Organization
